# Health literacy, health status and health behaviors of German students– study protocol for the “Healthy Habits” cohort study

**DOI:** 10.1186/s12889-021-11542-w

**Published:** 2021-08-06

**Authors:** Martin Lange, Alexandra Löwe, Gerrit Stassen, Andrea Schaller

**Affiliations:** 1 Department of Fitness and Health, IST University of Applied Sciences, Erkrather Straße 220 a-c, 40233 Düsseldorf, Germany; 2 Working Group Physical Activity-Related Prevention Research, Institute of Movement Therapy and Movement-oriented Prevention and Rehabilitation, German Sport University Cologne, Am Sportpark Müngersdorf 6, 50933 Cologne, Germany

**Keywords:** Students, Health literacy, Health status, Health behavior, Cohort study, Prevention, Digital health interventions, App

## Abstract

**Background:**

The emerging adulthood is traditionally viewed as a time of optimal health, but also as a critical life span, characterized by changing life circumstances and the establishment of an individual lifestyle. Especially university life seems to hold several challenges impeding the manifestation of a health supporting manner, as many students tend to show a poorer health behavior and a higher amount of health-related problems than comparable age groups. This, along with a steady growth of the higher education sector, brings increased attention to the university setting in the context of prevention.

To date, there are few empirical longitudinal and coherent cross-sectional data on the status of students’ health literacy, health status, and health behaviors, and on the impact of the study format on students’ health. The aim of this prospective cohort study is to reduce this research gap.

**Methods:**

Starting during winter semester 2020/21, the prospective cohort study collects data on health literacy, health status and health behavior on a semester-by-semester basis. All enrolled students of the IST University of Applied Sciences, regardless of study format and discipline, can participate in the study at the beginning of their first semester. The data are collected digitally via a specifically programmed app. A total of 103 items assess the subjectively perceived health status, life and study satisfaction, sleep quality, perceived stress, physical activity, diet, smoking, alcohol consumption, drug addiction and health literacy. Statistical analysis uses (1) multivariate methods to look at changes within the three health dimensions over time and (2) the association between the three health dimensions using multiple regression methods and correlations.

**Discussion:**

This cohort study collects comprehensive health data from students on the course of study. It is assumed that gathered data will provide information on how the state of health develops over the study period. Also, different degrees of correlations of health behavior and health literacy will reveal different impacts on the state of students’ health. Furthermore, this study will contribute to empirically justified development of target group-specific interventions.

**Trial registration:**

German Clinical Trials Register: DRKS00023397 (registered on October 26, 2020).

## Background

The emerging adulthood (age span of 18–25) is traditionally viewed as a time of optimal health with low levels of morbidity and chronic disease [1, 2]. At the same time, young adults appear to be more prone to psychosomatic health symptoms, depending on their individual life satisfaction and perceived future outlook [3, 4]. Characterized by changing life circumstances, personal growth and the manifestation of a certain lifestyle, the emerging adulthood is a distinct life phase [5, 6]. In comparison with other age groups, young adults tend to consume more alcohol, tobacco and drugs [7, 8]. Therefore, this life stage occurs as a vulnerable and critical time, in which specific health interventions might help paving the way for a healthy lifestyle. Especially university life can hold several challenges for students impeding the manifestation of a health supporting behavior [9].

On the one hand, the variety of study formats opens up considerable freedom for individually adaptable life concepts, such as studying alongside a part-time or full-time job, flexible lecture periods or studying during parental leave. The proportions on the spectrum from purely physical presence on site to exclusively digital forms of learning and examination from home can be selected according to the students’ individual life situation [10]. The university setting receives increased attention in the context of prevention, both because of the described health situation of students and a steady growth of the higher education sector [10]. Especially Universities of Applied Sciences (UAS) register an increasing number of students due to offering simplified access for professionally qualified persons, (study) flexibility and a high diversity of studies in the form of dual and part-time courses [10, 11].

On the other hand, this freedom and flexibility seem to come with a price. Changes in stress situations and strain parameters can be observed when it comes to meeting work and study requirements. Some studies identified factors such as double and multiple burdensome-situations, a disruptive study-family-balance, an uneven study-leisure-time-balance and severe work-related psychological stress situations [12–15]. Other requirements that students face during their studies include, for example, mastering demanding curricula, time-consuming workloads as well as mental and emotional challenges [16]. Current research of students’ health in Germany reveals an increased burn-out potential, an overall increased stress load, an above-average level of anxiety, sleep disorders, physical symptoms such as body aches or back pain and an overall subjectively lower-rated health status than comparable cohorts [12, 17–21]. As part of the HISBUS Panel, a large-scale cross-sectional study with a total net sample of *n* = 6198, female participants in particular reported physical and psychological complaints. Additionally, about 75% of the HISBUS cohort stated to suffer from physical complaints several times a month [17]. The students’ health status seems to reflect the consequences of permanent overload in diverse ways.

Studies indicate, that a poor state of health might result from the interaction of multiple factors, e.g., an insufficient health behavior or a low degree of health literacy [22]. The majority of studies pictures a linear relationship between the three health dimensions, stating that health literacy influences the health behavior of a person and thereby impacts health outcomes [23]. Contrary to that, some studies report a different constellation of the three health dimensions, where this linearity has not been observed at all or not even discover an association between health literacy and certain health behaviors, e.g. smoking health professionals [24, 25]. In fact, current studies on college students’ health behavior and health literacy point to a linear as well as reciprocal relationship. Accordingly, a linear view with only consecutive seems to fall short, for the dynamic of interactions, feedback effects as well as antecedents and consequences cannot be integrated [26]. Accompanying, external or social factors can increase the interaction of the health dimensions, influencing the state of health positively or negatively. With regard to health behavior, the above-mentioned stressors have a negative effect on the amount of students’ physical activity and nutritional behavior [17, 27, 28]. Drug and alcohol consumption have also been shown to increase among students [17, 29]. Although to interpret with caution, the HISBUS Panel [17] attested students a poorer health behavior in many aspects compared to non-students of the same age. In particular, the results revealed lower levels of physical activity, increased alcohol and nicotine use [29], abuses of cocaine and cannabis, as well as increased intake of painkillers [17].

In this context, health literacy is an important individual competence and related to an overall literacy. It includes knowledge, as well a set of cognitive, social and motivational skills, enabling people to access, understand, appraise, and apply health information [26, 30, 31]. Also, health literacy entails the capacity of making health-related judgements, taking decisions and establishing health-promoting behaviors on a daily basis (e.g., a healthy diet, physical activity, stress management) [32–34]. This understanding suggests, that health literate students are more likely to address the requirements and burdens described.

Despite the need of gaining more understanding of the complex nature of the relationship between the above-mentioned health dimensions, these studies also show different characteristics of the health dimensions among the students. This suggests the necessity of different approaches within the framework of possible health interventions.

Against this background, the aim of this cohort study is to gain insight in the relationship and change of UAS students’ health literacy, health status and health behaviors during their studies. Empirical inventories of student health differ both in their understanding of health and in the indicators collected [35, 36]. Thus, the cohort study’s assessment incorporates the broad categories of Dietz et al’s systematic umbrella review [36] to provide further clarification on the factors influencing student health (substance use, mental health/wellbeing, diet and nutrition, physical activity, sleep hygiene, media consumption and others). In this context, the following research questions will be addressed:

1. How do health behavior, health status and health literacy change during the course of study and after graduation (12 months post)?
2. What influencing factors on health behavior, health status and health literacy of UAS students can be identified?

## Methods / design

**Fig. 1:**
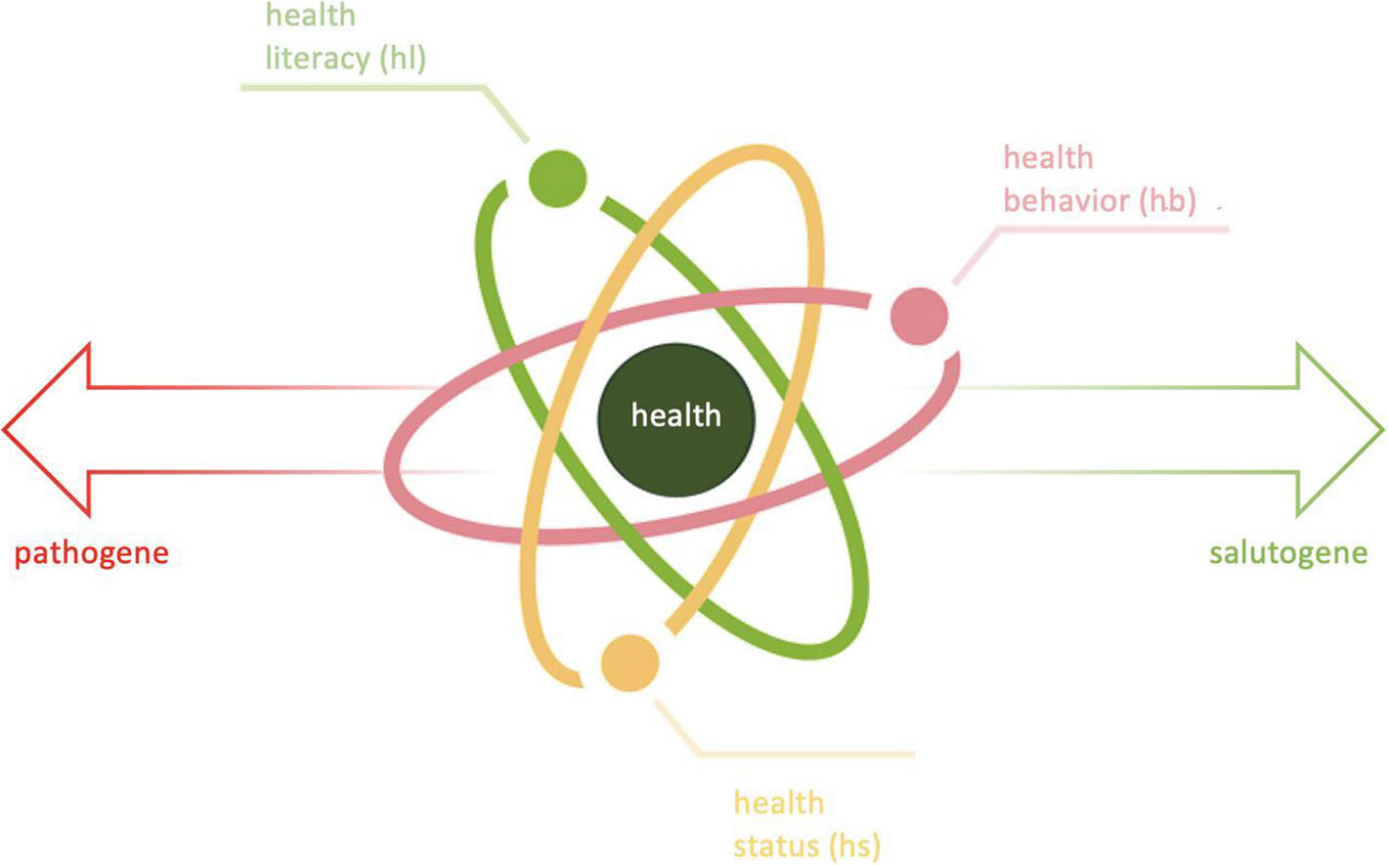
The multidimensional and dynamic construct of students’ health as the underlying construct

The German health promotion initiative “health-promoting university” is the overarching framework of the initiated *Healthy Habits* research project [37]. The cohort study is founded on a biopsychosocial and salutogenetic approach and assumes a multidimensional health continuum [38, 39]. If the salutogenetic approach is applied to the health of individuals, a three-way split emerges, where the state of health dynamically results from the aspects of health behavior as a generalized source of resistance and health competence as a superordinate empowerment in the sense of coherence. In summary, this leads to an understanding of health as a multidimensional and dynamically interacting construct, with the three core dimensions health status, health literacy and health behavior (see Fig. 1).

### Design of the study

**Fig. 2:**
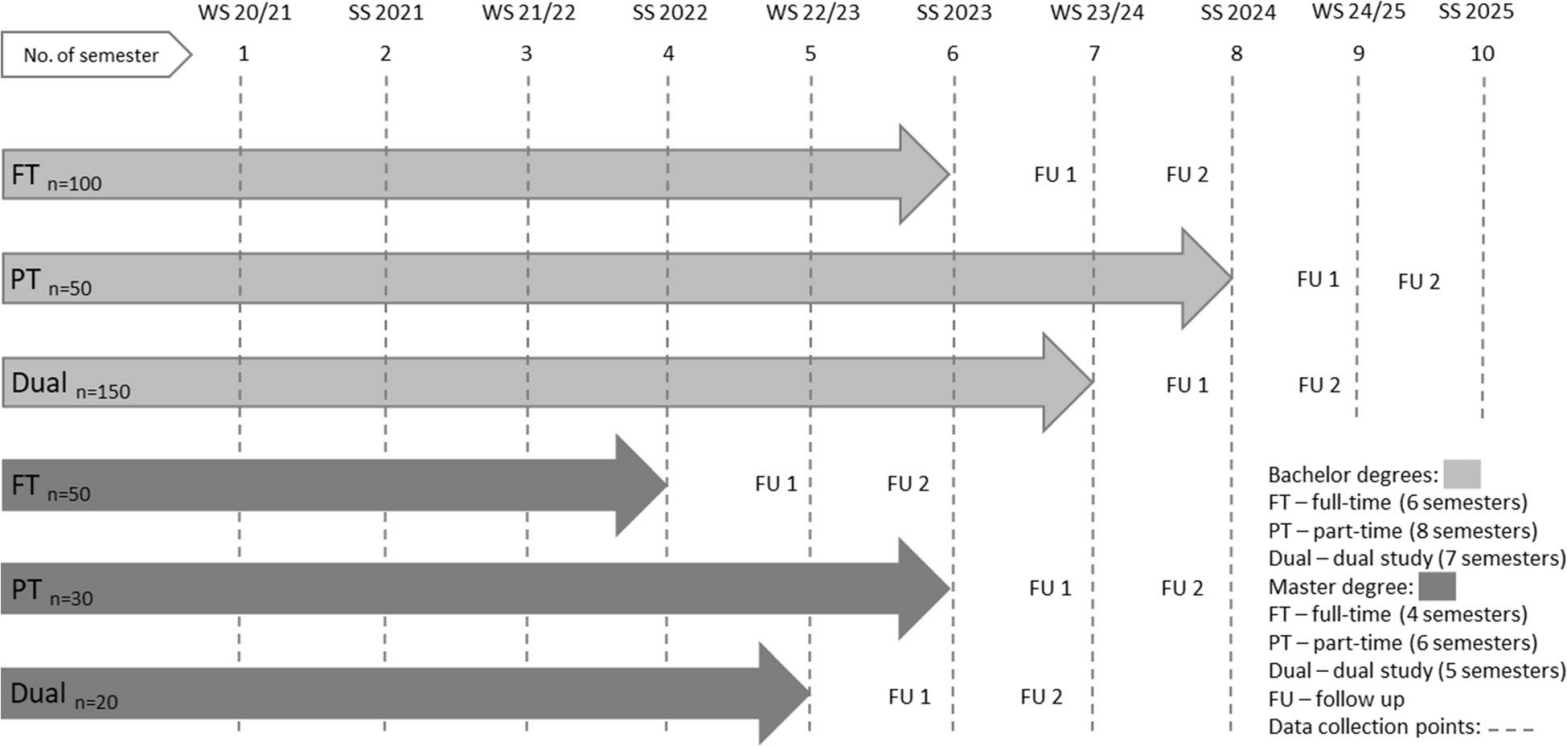
Study duration and assessment timeline for different Bachelor- and Master-degrees

The research design follows a longitudinal, prospective cohort study of enrolled UAS students at the IST University of Applied Sciences in Germany. STROBE (strengthening the report of observational studies in epidemiology) guidelines were applied in alignment with the research objective [40]. The frequency of data assessment is set to a semester-by-semester cycle (see Fig. 2). During the winter semester 2020/2021 the first semester students are being recruited for the first time.

### Sample and sample size

Students have been invited by email to participate in the cohort study and additionally have been introduced to the *Healthy Habits* project (official German website under https://healthyhabits.ist.de/) in several seminars at the beginning of the semester. The email contains information of the study, an invitation link to the research homepage and an identification code. The invitation email has been sent to all active and enrolled first semester students of all departments (sports business, fitness & health, tourism & hospitality, communication & business). Students, which have set their status to inactive (e.g. maternal break or personal matters) for more than one semester won’t be included. Since this is an exploratory cohort study no formal sample size calculation was done. We assume the participation rate of first-semester students to range from 20 to 40%. This would mean an average dataset of *n* = 400 per semester. This calculation is made defensively due to the constraints imposed by the Covid-19 pandemic.

### Data collection

Data is collected online using a questionnaire tool implemented in a progressive web application. This app is specially programmed for this research project. The questionnaire can be edited step by step, answers are saved automatically. There is no possibility to skip single items. After answering all questions, the students can submit their results and with that make no further changes.

Gathered data is stored on a separate server, taking into account current European as well as federal data protection security standards (DSGVO) in full. A connection to student records at the IST University of Applied Sciences is excluded, nor is the project team able to gain access to the user profile credentials.

### Variables under study and assessment

Health status, health behavior and health literacy are registered on the basis of different domains, for which a positive correlation with the respective health dimension could be determined.

Health-related quality of life, sleep quality, overall life satisfaction, self-perceived stress and self-perceived health status are seen as predictive measurements for health status [9, 41, 42]. To assess the dimension of health behavior the domains of health-related physical activity, screentime, nutritional behavior, alcohol consumption, smoking habits and drug consumption are referred to [43]. Health literacy is the only dimension which is validated as a construct itself and will therefore not be predicted through other surrogate constructs. Table 1 provides an overview of the selected constructs and the primary outcome parameters to operationalize the three health dimensions. To gather comparable data, the selection of variables was based as far as possible on similar studies on each of one of the three dimensions.

**Table 1:** Health dimensions and their associated constructs, selected references and primary outcome parameters

Content / Appropriateness	Instruments (Short form)	Primary Outcome Parameter (type of data)	Domains	No of Items	Validity	Reliability	Literature
**Health status**							
Self-rated health status: a concept widely used in European surveys; considered as appropriate marker for overall health status [44]	Minimum European Health Module (MEHM1)	Self-rated health status (interval)	General subjectively perceived health status	1	n/a	Internal consistency: α = .74	[44–47]
Quality of life (QoL): considered highly predictive to physical and psychological health status; widely used [48]	German version of the Satisfaction with life scale (SWLS)	Self-rated quality of life (interval)	Subjectively perceived life satisfaction	5	Convergent validity: *r* = −.34 to *r* = −.49	Internal consistency: α = .74	[49–51]
Satisfaction with life and studies: context-specific marker; comparability with existing data [17]	Life and study satisfaction scale (German titel: Lebens- und Studien-zufriedenheitsskala) (LSZ)	Self-rated life and study satisfaction (interval)	General feeling of efficiency, relationship to others, satisfaction with oneself and one’s own academic performance	7	Criterion validity: *r* = −.51; *r* = −.55;	Internal consistency: α = .79	[41, 52]
Perceived stress: suitable marker for mental health [53]	German version of the Perceived Stress Scale (PSS-10)	Self-rated stress (interval)	Subjectively perceived stress level	10	Construct validity: α = .95 to α = .96	Internal consistency: α = .84	[54]
Sleeping behavior and sleep quality: high predictive value of various parameters reflecting health status [55, 56]	German version of the Pittsburgh Sleep Quality -Index (PSQI-D)	Self-rated quality of sleep, sleep duration and interruptions (interval, ratio)	Self-assessment of sleep duration, efficiency and quality	19	Sensitivity and specificity: α = .80 to α = 1.00	Test-Retest: α = .89	[57]
**Health-related behavior**							
Physical activity: self-evaluation report of WRPA, TRPA, LRPA^a^ and strengthening training; high level of physical activity correlates positively various parameters reflecting health status [58, 59]	European Health Interview Survey - Physical Activity Questionnaire (EHIS-PAQ)	Self-reported: - METxHours - Sitting time - Intensity of activity (interval, ratio)	Physical activity and inactivity at different levels of intensity (MVPA, VPA^b^) and domains (WRPA, TRPA, LRPA^a^)	8	Convergent validity: *ρ* > .41	Test-Retest: ICC = .43 to ICC = .83	[60]
Screentime: usage duration, duration of use correlates negatively with various parameters reflecting health status [61, 62]	Screen Time (adapted to [55])	Duration of screentime (ratio)	Self-evaluation report of daily screentime; orientation to threshold values for children and adolescents	6	n/a	Test-Retest: ICC = .50 to ICC = .90	[63, 64]
Nutritional behavior: compliance with the dietary recommendations of the German Nutrition Association (DGE)	Nutrition; based on the Questionnaire for recording health behavior (German title: Fragebogen zur Erfassung des Gesundeitsverhaltens) (FEG)	Self-rated nutrition behavior (nominal, ordinal)	Self-evaluation report on the achievement of and compliance with the dietary recommendations of the German Nutrition Association (DGE)	13	n/a	n/a	[65]
Risk behavior (alcohol, smoking, addictive substances): self-evaluation report on risk thresholds for consuming critical substances (like nicotine, alcohol and addictive substances) [66, 67]	Brief Alcohol Screening Instrument in Medical Care (BASIC)	Amount and frequency of alcohol intake (nominal, ordinal)	Self-assessment on risk threshold for consuming alcohol	6	Diagnostic validity: Sensitivity α = .98; Specificity α = .88	Internal consistency: α = .81	[68]
	Smoking (based on FEG)	Amount and frequency of nicotine intake (nominal, ordinal, ratio)	Self-assessment on smoking behavior, smoking amount and duration of abstinence	1–3	n/a	n/a	[65]
	Substance (based on FEG)	Amount and frequency of drug intake (ordinal)	Self-assessment on risk threshold for consuming addictive substances, drug amount and duration of abstinence	7	n/a	n/a	[65]
**Health literacy**							
Health literacy: widely validated construct with multiple reference data pools of European cohorts [69]	European short form of the Health Literacy Survey (HLS-EU-Q16)	Self-rated health literacy (interval)	Self-assessment on health literacy	16	Convergent validity: *r* = .86	Internal consistency α = .78 to α = .97	[69–71]

^a^*WRPA* Work-Related Physical Activity, *TRPA* Transport (commuting) Physical Activity, *LTPA* Leisure-Time Physical Activities

^b^*MPVA* Moderate-to-Vigorous Physical Activity, *VPA* Vigorous Physical Activity

The assessment is composed of 10 established questionnaire-based instruments with a total of 101 items. As Table 1 shows five instruments are used to assess health status. Health behavior uses a total of four instruments. One instrument has been selected to assess health literacy.

To obtain a representative picture of students’ health status a single-item of the Minimum European Health Module (MEHM1), 5 items of the German version of the Satisfaction With Life Scale (SWLS), 7 items of the German Life- and Study-Satisfaction-Scale (LSZ), 10 items of the German version of the Perceived Stress Scale (PSS-10) and 19 items of the German version of the Pittsburgh Sleep Quality-Index (PSQI-D) are being included. All instruments show acceptable validity and reliability measures, offer reference values and are widely used to assess health status (see Table 1).

Health-related behavior covers a variety of behavioral domains and their measurement in large cohort studies is very complex. For the described research project, the domains of physical activity, screentime, nutrition, smoking habits as well as alcohol and drug consumption are of interest. Related data is collected by using 8 items of the Physical Activity section of the European Health Interview Survey (EHIS-PAQ), 6 items of the Brief Alcohol Screening Instrument in Medical Care (BASIC). Smoking habits (1–3 items), drug consumption (7 items) and nutrition behavior (13 items) is assessed with a total of 23 adapted items of the FEG-questionnaire (original: Fragebogen zur Erfassung des Gesundheitsverhaltens [Questionnaire to assess health behavior]). Non-smoking participants have to answer only 1 item and are led to the next domain. To measure time spent with digital devices 6 items of the self-rated Screen-time Questionnaire [63] were selected, modified and supplemented.

The 16-item European shortform of the health literacy Survey (HLS-EU Q16) concludes the assessment. The authors of this paper reviewed the critics of the original version of the HLS-EU [72] and therefore selected the latest updated shortform of the instrument. The published reference values as well as the statistical supported counter publication underline the benefits of the HLS [34].

For all instruments items’ content and answering format are used as published and have only been modified to fit the digital progressive web application.

### Statistical analyses

Descriptive statistics (mean, distribution standard deviation (SD), median, minimum, maximum, absolute and relative frequencies) will be conducted to describe the cohorts’ sociodemographic features (gender [male/female/diverse]; age [year of birth]) and study-related characteristics (type of degree [BA/MA], field of study [health-related studies vs. non-health-related] and study format [dual/part-time/full-time]). This stratified analysis will apply for all statistical analysis.

The changes in health behavior, health status and health literacy (research questions 1&2) will be each evaluated by means of variance analysis with measurement repetition. After checking the statistical model prerequisites, sociodemographic and study-related influencing factors on health behavior, health status and health literacy will be each tested by means of linear regression analysis.

For all calculations the level of statistical significance will be set to *p* < 0.05 [73] and SPSS® (Statistical Package for the Social Sciences, IBM, Version 27) will be used.

## Discussion

Attending a university or UAS is a lifechanging event in general and can be a very formative phase of life for young adults. Students will learn to deal with stress, the burden of learning for exams, setbacks as well as successes and overall to take responsibility for themselves. Unfortunately, taking care of one’s own health is not always priority number one during that phase of life. Current studies provide indications that students show a poor health behavior [17, 29]. The overall consequences of an unhealthy lifestyle as well as the insufficient management of psychophysical requirements are not only reflected in a poorer state of health, but also have an impact on the course of the study. Lower academic performance, a significantly longer duration of study and even drop-outs are possible consequences [16, 74]. According to the German Center for Higher Education and Science Research (original: Deutsches Zentrum für Hochschul- und Wissenschaftsforschung [DZHW]) the dropout rate ranges between 15 to 35% depending on the type of study and the subject [75].

To address these aspects efficiently and sustainably with interventions, requires a further understanding of how health changes during the course of study as well as of the impact of influencing factors. A mere consideration of health status does not fulfill the complexity, since it is not always known whether a poor health status results from an insufficient health behavior or a lack of competence. Recent research shows that only about 30.3% of students have sufficient health literacy [76]. There are also significant differences between male and female students. Furthermore, students with a migrant background as well as students with lower degrees (bachelor’ degrees) and first semester students have significantly poorer health literacy [77–79]. These studies also suggest the existence of different target groups within the setting of UAS students which in turn should be approached differently with tailored interventions. To the authors best knowledge such comprehensive studies have not been sufficiently conducted yet in an UAS setting.

Contrary to growing scientific interest in student health research in recent years, the current amount of data is consistently inadequate. Most of the existing studies either looked at the three health dimensions separately from each or are mostly based on cross-sectional examinations [9, 17, 20, 41]. Longitudinal studies on the three health dimensions over the course of the study, on the other hand, are rare. Also, the quantity and quality of studies investigating the association between the described health dimensions and their mutual influence among themselves within the setting of students are insufficient as well.

Despite the mentioned promising potential of the *Healthy Habits* research project, field research challenges as well as limitations have to be mentioned. In consequence of the Covid-19 pandemic the starting of participants’ recruitment had to be postponed to December 2020. In addition, as a result of federal restrictions all in-person seminars are prohibited, so that for the entire winter semester 2020/2021 only online-based seminars are offered. First semester events such as initiations and other in-person inauguration seminars have been canceled. Therefore, the communication with the students can only take place digitally.

Another potential distortion can be caused by assessment. After completing the app-based questionnaire, the results are displayed in form of a radar chart. Each health dimension is displayed separately, reflecting aspects of the selected assessment instruments. The authors are aware of the fact, that receiving an evaluation of one’s questionnaire responses might be seen as a first health intervention, increasing students’ awareness for health topics. The overarching intention is to motivate students to participate in the assessment sustainably.

The *Healthy Habits* research project major strengths are the longitudinal design and the app-based approach to reach a more and more digital affine target group. This mainly digital approach widens the spectrum of possible interventions, which also varies by format, content and degree of individualization. Fields of actions (original: Handlungsfeld) are legally defined areas in which preventive interventions have to take place, including physical activity, diet, stress and addiction. Next to classic course interventions, additional formats may include gamification elements such as challenges or quizzes, push-up messages, podcasts, blogs, webinars or scribble videos. Also, it is possible to address subgroups or single individuals of the target group by assigning achieved assessment scores to certain interventions. The findings will bring greater understanding of how to address student’s challenges with tailored preventive interventions.

## Data Availability

The datasets used and/or analysed during the study will be available from the corresponding author on reasonable request.

## Abbreviations

UAS: University of Applied Science
WS: Winter Semester
SS: Summer Semester
BA: Bachelor
M: Master
SD: Standard deviation
MEHM1: Minimum European Health Module
SWLS: German version of the Satisfaction With Life Scale
LSZ: Life- and Study Satisfaction Scale (original: Lebens- und Studienzufriedenheitsskala)
PSS-10: German Version of the Perceived Stress Scale
PSQI-D: German version of the Pittsburgh Sleep-Quality-Index
EHIS-PAQ: European Health Interview Survey - Physical Activity Questionnaire
FEG: Questionnaire to assess health behavior (original: Fragebogen zur Erfassung des Gesundheitsverhaltens)
BASIC: Brief Alcohol Screening Instrument in Medical Care
HLS-EU-Q16: European shortform of the Health Literacy Survey
MPVA: Moderate-to-Vigorous Physical Activity
WRPA: Work-Related Physical Activity
TRPA: Transport (commuting) Physical Activity
LTPA: Leisure-Time Physical Activities
